# Ontogeny of alkaline phosphatase activity in infant intestines and breast milk

**DOI:** 10.1186/s12887-018-1379-1

**Published:** 2019-01-03

**Authors:** Ye Yang, Emilee Rader, Michele Peters-Carr, Rebecca C. Bent, Jennifer T. Smilowitz, Karen Guillemin, Bethany Rader

**Affiliations:** 1 Institute of Molecular Biology, University of Oregon, Eugene, OR USA; 2 Present Address: Department of Medicine, University of Florida, Gainesville, FL USA; 3 Department of Media and Information, Michigan State University, East Lansing, MI USA; 4 PeaceHealth Nurse Midwifery Birth Center, Springfield, OR USA; 5 Neonatal Intensive Care Unit, RiverBend Medical Center, Springfield, OR USA; 6 Foods for Health Institute, University of California at Davis, Davis, CA USA; 7 Department of Food Science and Technology, University of California at Davis, Davis, CA USA; 8 Department of Microbiology, Southern Illinois University, Life Science II Room 131, 1125 Lincoln Drive, Carbondale, IL 62901 USA

**Keywords:** Necrotizing enterocolitis (NEC), Meconium, LPS detoxification, Gestational age

## Abstract

**Background:**

Necrotizing enterocolitis (NEC) is a devastating disease of intestinal inflammation that primarily affects premature infants. A potential risk factor for necrotizing enterocolitis is exposure of the premature neonatal intestine to environmental bacteria and their proinflammatory products such as lipopolysaccharide. The metalloenzyme alkaline phosphatase (ALP) has been shown to reduce lipopolysaccharide-mediated inflammation. Additionally, premature rat pups have reduced alkaline phosphatase activity and expression as compared to full term pups. To explore the possibility that the human premature neonatal intestine has a paucity of alkaline phosphatase activity, we measured endogenously produced intestinal alkaline phosphatase activity in meconium as a function of gestational age. To test whether breast milk could serve as a source of exogenous alkaline phosphatase to the neonatal intestine through ingestion, we measured alkaline phosphatase activity in breast milk across a range of time points post-birth.

**Methods:**

Alkaline phosphatase activity was quantified in 122 meconium samples from infants of gestational ages ranging from 24 to 40 weeks and in 289 breast milk samples collected from 78 individual mothers between days 2–49 post-birth.

**Results:**

We observed a strong positive correlation between the meconium alkaline phosphatase activity and gestational age, with preterm infants having lower meconium alkaline phosphatase activities than early term or term infants. Breast milk alkaline phosphatase activity was highest in the first week post-birth, with peak alkaline phosphatase activity at day 2 post-birth, followed by relatively low alkaline phosphatase activity in weeks 2–7.

**Conclusions:**

Our results are consistent with the two major risk factors for necrotizing enterocolitis development, preterm birth and lack of breast milk feeding, both contributing to a paucity of alkaline phosphatase activity and impaired capacity to detoxify proinflammatory bacterial products such as lipopolysaccharide.

## Background

Infants born prematurely are at risk of developing necrotizing enterocolitis (NEC), a multifactorial disease characterized by overly exuberant inflammatory responses in the immature intestine and a leading cause of late mortality and morbidity in very preterm infants [1, 2]. As there is no known cure for NEC, current research on the disease is in part focused on identifying risk factors for disease development in neonates with the eventual goal of identifying new treatment options and preventing the disease [3]. It has been suggested that the aberrant inflammation associated with NEC is due in part to the inability of the immature intestine to adapt to the premature establishment of the microbiota [4, 5]. After birth, microbes rapidly colonize the newborn intestine and introduce numerous antigens and toxins including endotoxin, or lipopolysaccharide (LPS), a constituent of the Gram-negative bacterial cell wall. LPS binds to the innate immune receptor Toll-like receptor 4 (TLR4) and induces inflammatory responses [6, 7]. Increased levels of LPS/TLR4 signaling have been proposed to contribute to the pathogenesis of NEC [4, 8–11]. Indeed, bacterial colonization has been identified as a primary risk factor for development of NEC in preterm infants [3, 12].

Alkaline phosphatases (ALPs) are conserved metalloenzymes that hydrolyze the release of inorganic phosphates from a variety of substrates [13]. ALPs have been shown to “detoxify” Gram-negative bacterial LPS by removing phosphates from the lipid A moiety, thereby decreasing its stimulation of TLR4 [14–18]. ALPs are found in a wide range of human tissues, including the gastrointestinal tract where ALP protein is localized to the apical membrane of enterocytes and enters the lumen through the secretion of microvillar vesicles [13, 19]. Interestingly, a dynamic transition of ALP isozyme forms is associated with the maturation of fetal intestines [13], suggesting that ALP activity may change during human fetal development. Furthermore, supplementation of the neonate rat pup intestine with ALP was protective against both LPS induced inflammation and experimentally induced NEC [20, 21], These data, in conjunction with a recent report showed that prematurity in rat pups was associated with reduced intestinal ALP expression and activity [22], identify ALP deficiency as a risk factor for the development of NEC in premature infants, however there are no studies to date reporting ALP activity of the developing human intestine as a function of gestational age.

ALP is also a known component of breast milk [23–27], and implicated as an anti-inflammatory factor in the newborn intestine [28]. Previous studies of ALP in breast milk have suggested a trend of decreasing ALP with time post-birth, but these studies have only surveyed small numbers of samples or limited time points [29–32]. In this study, we hypothesized that ALP activity in the infant intestine increases with gut maturation, and that a lack of ALP, and thus insufficient LPS detoxification, could contribute to the increased susceptibility of preterm neonates to NEC. Additionally, we hypothesized that ALP content in breast milk would be highest at earlier lactation stages when it would serve to supplement the infant intestine with LPS-detoxifying activity during the initial period of intestinal colonization by microbes. Using ALP activity as a proxy for ALP content, we conducted two separate studies, first characterizing the ALP activity in meconium samples from infants at different gestational ages, and second characterizing ALP activity in breast milk from seventy-eight mothers of full-term infants not associated with the previous study at different time points post-birth.

## Methods and materials

### Patients and meconium and breast milk samples

The use of meconium samples for this study was reviewed by the University of Oregon Institutional Review Board and Research Compliance Services and determined to qualify for an exemption as per Title 45 CFR Part 46.101 (b). A total of 122 meconium samples from infants of gestational ages ranging from 24 to 40 weeks (except 30 weeks) were obtained from the Peacehealth Neonatal Intensive Care Unit and the Peacehealth Nurse Midwifery Birth Center (Springfield, OR). Samples were frozen at − 80 °C upon collection and subsequently analyzed. A total of 289 frozen milk samples were collected on post-birth days 2–49 from 78 individual mothers who had given birth to term infants enrolled in the UC Davis FFHI Lactation Study [16, 33, 34]. Colostrum and breast milk samples were collected by hand expression from one breast by the trained participant and frozen immediately in participants’ homes and transported to the lab on ice and stored at − 80 °C. Samples were de-identified to protect patient privacy and ensure blinding during the ALP analysis. The University of California Davis Institutional Review Board approved all aspects of the study and informed consent was obtained from all subjects. Analysis of breast milk ALP was approved by the University of Oregon Institutional Review Board and Research Compliance Services (protocol #11052013.003). This trial was registered on clinicaltrials.gov (ClinicalTrials.gov Identifier: NCT01817127).

### Analysis of ALP activity in infant meconium

Meconium samples were homogenized in double distilled water and centrifuged at 16,000 g for 15 min at 4 °C to collect the supernatants. The supernatants were diluted and then assayed for protein concentrations using the Bio-Rad protein assay kit (Bio-Rad Laboratories Inc.) and for ALP activities using the PNPP substrate kit (Thermo Fisher Scientific Inc.). ALP activities were compared to standard shrimp alkaline phosphatase (SAP) (Thermo Fisher Scientific Inc.) and normalized to meconium protein concentrations. Data were grouped by weeks of completed gestation at birth, and analyzed using the Prism software (GraphPad software). Correlation between the meconium ALP activity and gestational age was analyzed using one-way ANOVA, posttest for linear trend. Meconium ALP activities in preterm newborns (gestational age ≤ 36 weeks), early term (37–38 weeks) and term (39–40 weeks) were compared using one-way ANOVA, followed by Bonferroni’s Multiple Comparison Test.

### Analysis of ALP activity in breast milk

To assay for ALP activity in breast milk, we modified a fluorometric detection method already published [35, 36]. Briefly, the samples were thawed, then vortexed to re-incorporate any separated cream, and diluted 1:10 in 100 mM Tris, pH 9.5. 50 μl of diluted sample was added to 50 μl of 2.5 mM 4-methylumbelliferone phosphate (4MUP) substrate in 100 mM Tris, pH 9.5 in a 96-well plate. Samples were incubated at room temperature for 5 min and then fluorescence was detected at 460 nM (excitation at 355 nM) using a FLUOstar Omega microplate reader (BMG Labtech, Cary, NC). Negative controls were sample wells with 4MUP and Tris alone and milk samples heated at 100 °C for 5 min to inactivate endogenous ALPs. All samples were analyzed in triplicate. To calculate ALP content, all ALP activity measurements were compared to a standard curve using shrimp alkaline phosphatase (Thermo Fisher Scientific Inc.). Sample data from one individual was removed from the data set as the first weeks measurement was three standard deviations away from the mean. Data for week 1, week2, weeks 3–4 and weeks 6–7 were analyzed with R version 3.3.2, with package ‘lme4’ version 1.1.13 and package ‘lmerTest’ version 2.0.33, using a mixed-effects regression with ALP activity as the dependent variable and week of sample collection as a fixed effect, categorical predictor and participant as the random effect. The intercept in this model (coefficient = 6.423, SE = 0.258) represents the level of ALP activity in the first week. The remaining model coefficients represent the difference between the ALP activity in the first week and ALP activity in Week 2, Week 3–4, and Week 6–7. The standard error for all coefficients was 0.33, and all coefficients in the model are statistically significant at the *p* < 0.001 level. This model is a better fit for the data than a baseline model having only the random effect for mother (F = 61.018, df = 3214; p < 0.001). A Levene’s Test of equality of variance in the samples collected in the four sampling windows was statistically significant, F(3,281) = 18.882, p < 0.001, indicating that the variances are not equal. A second linear regression model analyzed ALP content by day during the first week, with ALP activity as the dependent variable and day of sample collection as a categorical predictor. Only one sample per mother was collected during the first week, so this model does not include a random effect for participant. The intercept (coefficient = 10.198, SE = 0.884) represents the level of ALP activity in day 2.

## Results

### Meconium ALP activity is positively correlated with gestational age

**Fig. 1:**
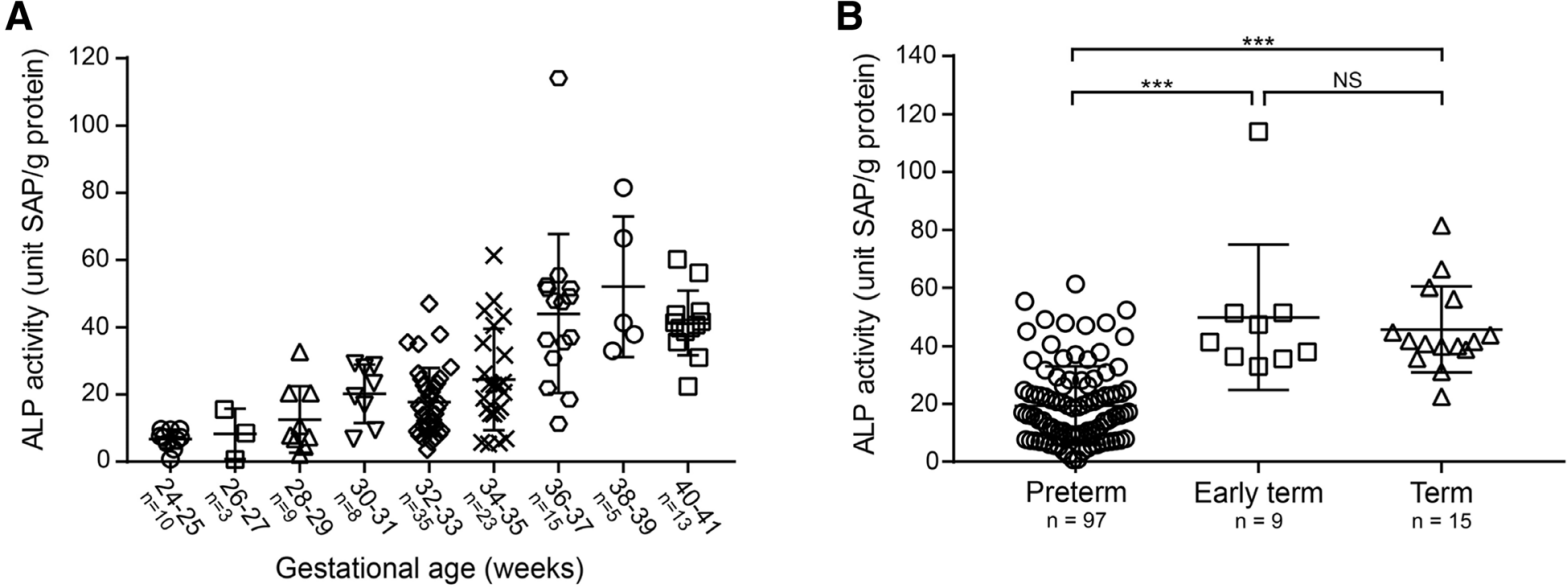
(**a**) Meconium ALP activity increases with gestational age (*P* < 0.0001, R^2^ = 0.3416; one-way ANOVA and posttest for linear trend). (**b**) Meconium ALP activities are significantly lower in preterm (up to 36 weeks of gestation) infants than those in term (37–38 weeks) or term (39–40 weeks) infants. (***, P < 0.0001; one-way ANOVA followed by Bonferroni’s Multiple Comparison Test). Error bars represent standard deviations, n is sample size

To investigate the amount of ALP in the infant intestine, we quantified ALP activity in meconium samples collected from infants at gestational ages 24–40 weeks. We observed a strong positive correlation between the meconium ALP activity and gestational age (*P* < 0.0001, R^2^ = 0.3416; one-way ANOVA and posttest for linear trend) (Fig. 1 A). The average meconium ALP activities from preterm (up to 36 weeks of gestation), early term (37–38 weeks of gestation) and term (39–40 weeks of gestation) infants (term definitions as reported in [37]) were determined to be 19.34, 49.85 and 45.64 units ALP/g protein, respectively. The preterm infants had significant lower meconium ALP activities than early term or term infants (P < 0.0001; one-way ANOVA followed by Bonferroni’s Multiple Comparison Test) (Fig. 1 B).

### Breast milk ALP activity is inversely correlated with days post-birth

**Fig. 2:**
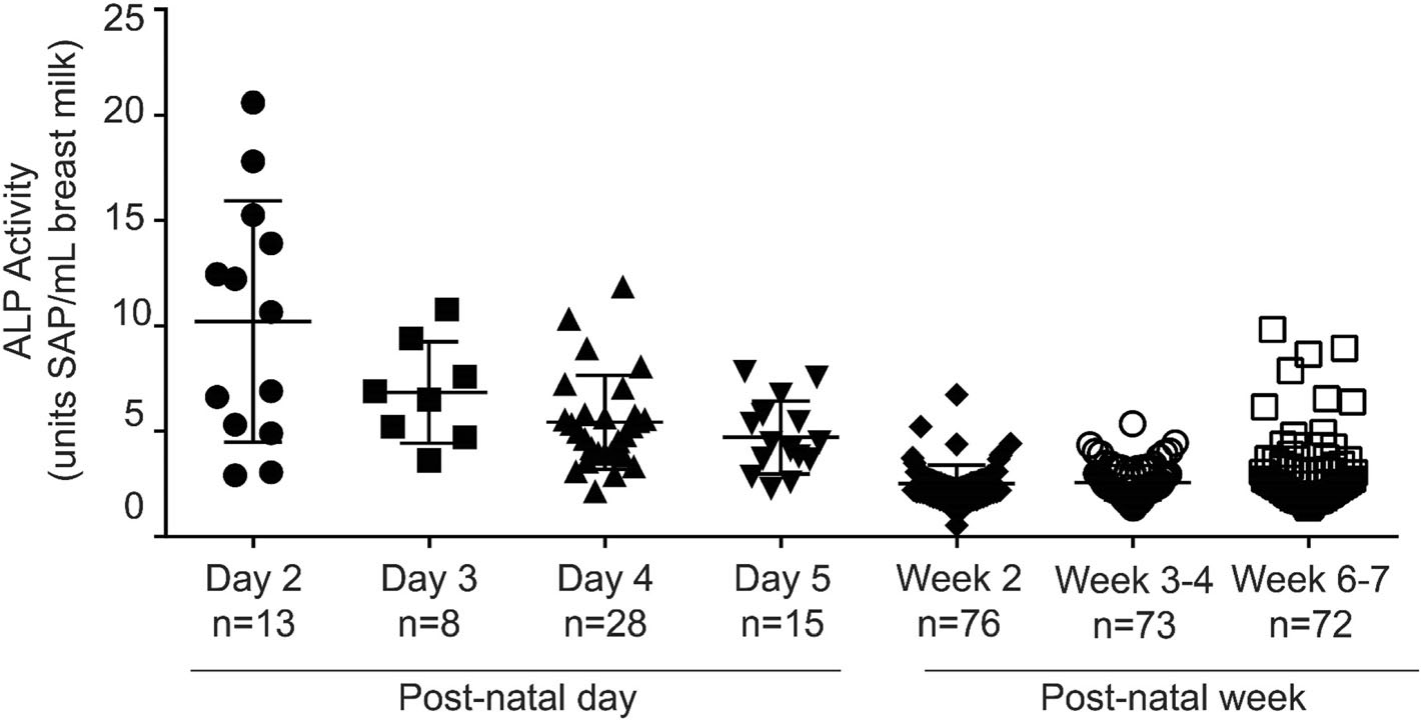
Alkaline phosphatase activity of breast milk as a function of time. Average ALP units in breast milk samples obtained in postnatal days 2–5, week 2 (days 8–13), week 3–4 (days 17–22) and week 6–7 (days 40–49). Error bars represent standard deviation and n is sample size. All coefficients in the model are statistically significant at the *p* < 0.001 level using a mixed-effects regression model in which model coefficients represent the difference between the ALP activity in the first week and ALP activity in week 2, week 3–4, and week 6–7

We quantified ALP activity in serial breast milk samples from women at day 2 post-birth though week 40 post-birth. We found that the absolute amount of ALP activity varied extensively among individual mothers. Despite this inter-individual variation, we found that breast milk sampled in the first week post-birth had 250% more ALP activity on average (6.40 units) than breast milk sampled in week 2 (2.50 units). A linear hypothesis test on the regression model coefficients showed that the difference between week 1 and week 2 is statistically significant (Wald χ2 = 139.912, df = 1, *p* < 0.001). Linear hypothesis tests comparing week 1 with weeks 3–4 and weeks 6–7 were also statistically significant (week 1 v. 3–4: Wald χ2 = 133.523, df = 1, *p* < 0.001; week 1 v. 6–7: Wald χ2 = 100.168, df = 1, p < 0.001). ALP activity remained within 25% of the week 2 average through weeks 3–4 (2.57 units) and weeks 6–7 (3.08 units) (Fig. 2), and comparisons between week 2 versus weeks 3–4, and weeks 3–4 versus weeks 6–7 were not statistically significant.

**Fig. 3:**
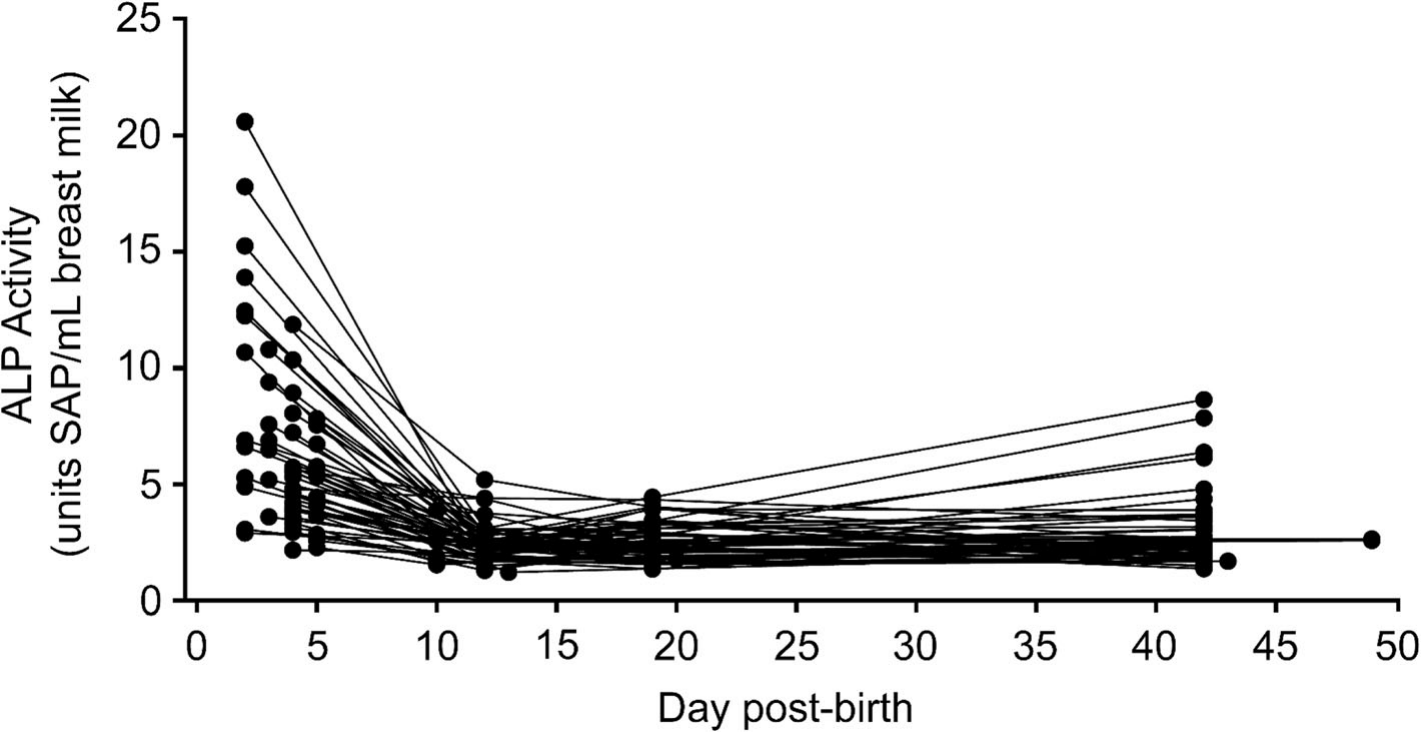
Trends in alkaline phosphatase activity by individual. Lines represent trends in ALP activity in serial breast milk samples from the 56 individual donors for which 4 milk samples were available. Each dot represents ALP units from each of the 4 individual milk samples, one from postnatal week 1, week 2, week 3–4, or week 6–7. Sample sizes are as follows, day 2, *n* = 13; day 3, *n* = 7; day 4, *n* = 23; day 5, n = 13; day 10, n = 7; day 12, *n* = 47; day 13, n = 2; day 17, n = 2; day 19, *n* = 50; day 20, n = 2; day 21, n = 2; day 42, *n* = 53; day 43, n = 1; day 49, n = 1

Within the first week, the earliest samples, collected at day 2 post-birth, had higher ALP activity than those collected on days 3, 4, and 5 (Fig. 2). The coefficients for day 3, day 4 and day 5 are all negative and statistically significant (day 3: coefficient = − 3.362, SE = 1.432, *p* < 0.05; day 4: coefficient = − 4.774, SE = 1.07, p < 0.001; day 5: coefficient = − 5.492, SE = 1.208, p < 0.001). All individuals for whom we had samples in all 4 time intervals displayed the same trend of decreasing ALP activity over time (Fig. 3).

## Discussion

In this study, we discover for the first time that premature infants have reduced intestinal ALP activity at birth as compared to early term or term infants (Fig. 1). These results are consistent with a recent study in premature rat pups [22] and provide a possible contributing factor in the etiology of NEC, a devastating disease impacting 12% of very low birth-weight infants [38]. After birth, intestinal ALP activity is likely to be up-regulated by environmental factors such as microbes and food components [17, 39]. The ALP deficiency we have documented in the preterm newborn intestine would be expected to impact early innate immune responses to bacterial colonization of this organ. While our study is limited in scope compared to the multifactorial nature of NEC, we propose that the reduced capacity of preterm infant intestines to dephosphorylate pro-inflammatory LPS may lead to excessive inflammatory responses to bacteria and thus increase the risk for developing NEC. Consistent with our prediction, analysis of the transcriptional profiles of intestinal tissue from NEC and control infants, as well as those of an experimental mouse model of NEC, revealed LPS as the top predicted upstream regulator of the NEC-specific profiles [40]. A corollary of our prediction is that early gut microbial communities high in LPS-containing Gram-negative bacteria would be another risk factor for NEC. Indeed, several studies have identified high levels of the Gram-negative phylum of Proteobacteria in infant stools as a hallmark of the onset of NEC [41-44].

In addition to endogenously produced ALP, infants who are breast fed or fed non-pasteurized donor breast milk may receive exogenous ALP. ALP is a reported component of breast milk, and a review of literature in which ALP activity is quantified in breast milk suggests a decreasing trend in ALP activity over time post-birth [23–25]. In addition, several studies have demonstrated that breast milk reduces susceptibility to NEC in comparison to formula or a combination of breast and bovine milk [45]. In fact the 2012 American Academy of Pediatrics policy statement recommended the use of human milk for preterm, term or other high risk infants [46]. We reasoned that breast milk may be designed to supplement ALP activity and provide protection against LPS-mediated inflammation to the neonatal intestine during the critical window of bacterial colonization that occurs during the first few days after birth. Therefore a second objective of our study was to characterize in detail ALP levels in breast milk as a function of time post-birth.

Our data demonstrate high levels of ALP activity in breast milk in the first few days post-birth, with a rapid decrease after the first week (Fig. 2). This early time period corresponds to the initial colonization of the naïve infant intestine by environmental microbes, including LPS containing Gram-negative bacteria. By day 2 post-birth, infants can have a dense microbial community [16, 47]. Our data therefore supports the hypothesis that high levels of ALP in breast milk may be one of many factors that promote tolerance to the high load of LPS experienced by the naïve infant intestine during initial colonization, prior to the infant’s endogenous innate immune system’s up-regulation of tolerance promoting mechanisms. Although the trend of highest ALP activity in week 1 breast milk is consistent across all samples, there is considerable between-mother variation in the amount of ALP activity (Fig. 3). We speculate that these inter-individual differences, which could be due to both genetics and environmental factors such as maternal diet and immune status, contribute to the infant’s overall resistance to intestinal inflammation upon initial colonization after birth. If ALP contributes to this protection, our data suggests that donor breast milk from pooled postpartum ages would be unlikely to contain significant amounts of this enzymatic activity prior to pasteurization. Donor milk is mainly pasteurized using heat at temperatures that will inactivate ALP activity as well as other bioactive milk components [48]. To compensate, both donor milk and mother’s milk, which may lack some of these components due to natural decrease correlated with post-partum date of expression, are often fortified with commercially available pre-term or low birth weight formulas [49]. However, to our knowledge, these formulas to not contain ALP specifically. In addition, many premature infants are exclusively fed intravenously until feeding tolerance is determined clinically [50]. It is therefore unlikely that pre-term infants not receiving mother’s milk within the first week of life will receive appreciable amounts of exogenous ALP.

## Conclusions

Our findings suggest that there is a coordinated mother-infant program of defense against the pro-inflammatory insults of intestinal bacterial colonization that occurs after birth. We hypothesize that the higher ALP activity in the full-term neonatal intestine, combined with high ALP activity of breast milk within the first few days post birth, provides adequate capacity to detoxify the LPS of initially colonizing bacteria. We suggest that paucity of this activity in the preterm intestine and in the absence of early post-birth breast milk feeding, increases the risk of excessive inflammation and progression to NEC. A limitation of our study is that we did not have meconium and breast milk samples from mother-infant pairs. We anticipate that our initial findings will motivate prospective studies on the interplay between intestinal ALP, breast milk ALP, gut microbiota, and NEC development in premature infants. Such studies may generate support for prophylactic ALP supplementation to premature infants as an effective therapeutic for NEC prevention.

## Abbreviations

**ALP**: alkaline phosphatase
LPS: lipopolysaccharide
NEC: necrotizing enterocolitis
TLR4: toll-like receptor 4
